# Rambles in Military Hospitals

**Published:** 1864-04

**Authors:** R. M. Lackey

**Affiliations:** M. D., A. A. Surg., U. S. A.


CHICAGO

### MEDICAL JOURNAL.

VOL. XXI.] APRIL, 1864. [No. 4.

### ORIGINAL COMMUNICATIONS.

### RAMBLES IN MILITARY HOSPITALS.

PRISON HOSPITAL, ROCK ISLAND, ILL.

By R. M. LACKEY, M.D., A. A. Surg., U. S. A.

After a twelvemonth's rest, I once again resume my "Rambles," and this time I greet you from a different latitude from that in which I made my last excursion. This time I am not in Dixie, but where quite a large number of her deluded sons find a temporary abiding place.

The island upon which this prison is located, and long known as Rock Island, is situated in the Mississippi River, in latitude, north, about 40 degrees. Opposite the lower end of the island is the city of Rock Island, on the Illinois side, and Davenport on the Iowa side of the river. The island is high, and the surface rolling; the soil sandy, with gravelly, rocky subsoil. A large portion of it is covered with a growth of fine young timber, the larger timber having been nearly all cut off. The island contains about 1000 acres, nearly all of it belonging to Uncle Sam.

The only buildings of much importance on the island, except those erected recently by the government, are those built by Col. Davenport in 1839. The dwelling house in which he was murdered is a commodious, substantial frame building, and is now occupied by the Post Commandant, Col. Johnston, as his headquarters.

The barracks are built on a ridge running parallel with the river, near the bank on the north side of the island. The buildings are like those usually erected for such purposes, they have been hastily put up, and of green, wet lumber, so that after a few days' seasoning, the ventilation is ample.

There is excellent opporturnity for drainage, but, unfortunately, from want of a little skill and foresight, or rather, I should say, for want of common sense in planning, there is not much gained by draining, as the engine and reservoir by means of which the place is supplied with water from the river, is placed below where the filth runs into the river, thus rendering the water used quite impure. But it is the medical department about which I desire to write more especially. The hospital was first organized by Dr. Thos. J. Ilis, of Davenport, Nov. 14th, 1863. There was then only four companies of the Invalid Corps here, but soon after 1,500 prisoners arrived, and of this number 10 per cent. were sick and subjects for hospital. No hospital had been erected, and a sufficient number of the barracks were appropriated for hospital purposes. Most of these men were sick with colds and pneumonia, contracted on the trip northward; they were poorly clad, and had been for a long time, probably, poorly fed, and consequently had but little stamina to resist the effects of cold. There was 327 sick prisoners admitted into hospital from Dec. 6th to Dec. 20th, 1863, and according to the record kept, there was of this number 189 sick with pneumonia. The others were mainly old cases of diarrhœa, debility, and acute rheumatism. The number of deaths during that time was 38, and of these 32 died of pneumonia, and only 6 from other diseases. It will be seen that the cases of pneumonia were of a severe grade, for of the whole number admitted to hospital about 97 per cent. died of this disease alone, and of the whole number of cases about 17 per cent., and undoubtedly quite a number of those admitted during that time with pneumonia, died subsequently.

There is not so large a proportion of pneumonia cases now as previously, when there were more fresh arrivals of prisoners; those that do occur are mostly among those newly arrived. It is scarcely necessary for me to speak of the treatment pursued in these cases, as it is evident to every intelligent medical man, that it must be mainly supporting, by the admininistration of tonics, stimulants, and nourishment; these will not bear sedatives or depletion in scarcely any case.

The cases of rheumatism are usually treated with palliatives, a Dover's powder, 10 grs., at bed-time, and stimulating and anodyne liniments locally.

Variola is now the predominationg disease among the prisoners. There are at this time 771 patients in the prison hospital, and fully one-half this number are in the pest-house. There are a few cases of Camp Fever, and but few in proportion to the large number of men that are crowded together, and many of them exceedingly filthy in their habits. There is a strong tendency to erysipelas and hospital gangrene, so that the few cases of wounds that we have are exceedingly troublesome. The slightest abrasion of the skin often results in a dangerous erysipelatous inflammation.

Iodine is the remedy most used as a local application, and I am more than ever convinced that there is nothing better now in use; even in the so-called "hospital gangrene," I prefer it to Bromine. When a suppurating surface presents that condition we call "gangrenous," I at once apply a strong solution or the officinal Tinct. of Iodine, twice a day, and usually after a few applications I have the satisfaction of seeing the wound assume a healthy appearance, and Bromine can do nothing more.

There are between 8, and 9,000 prisoners here, and up to this time, there has been about 1,900 sent to the hospital, and the whole number of deaths has been about 575. This shows a fearful rate of mortality, for the number of hospital cases.

Small Pox appeared among the prisoners first about the 1st of Jan., 1864. The disease was brought here by a lot of prisoners from Tenn., some of whom had been exposed to the contagion. From the first, the disease made rapid progress notwithstanding the medical officers made every effort in their power to stay its ravages. They insisted on the necessity of isolating the cases as they occurred, and protested against any more prisoners being sent here until the disease abated. But it was in vain in this, as in many other cases, for medical officers to try to accomplish anything without the co-operation of other officers.

It was some time before buildings were erected for a "pest-house," and even now with about 400 cases of small pox, there is only hospital accommodations for about 200, in a proper locality, and fresh installments of prisoners are arriving almost daily, and as a consequence the disease is fearfully on the increase. The mortality is proportionately greater now than when the disease first appeared, showing that from concentration of a large number of cases the virulence of the disease is increased.

A large amount of the "Saracenia purpura" has been used in the treatment of small pox here, and so far it has failed to have the beneficial effects that it is said to produce in variola. It is being fairly tested here, and it is hoped that it may prove efficacious. There is but little sickness in the garrison at present; a few cases, only, of small pox have occurred amongst our own men. But I have rambled far enough for once.

